# Development and validation of an epitope prediction tool for swine (PigMatrix) based on the pocket profile method

**DOI:** 10.1186/s12859-015-0724-8

**Published:** 2015-09-15

**Authors:** Andres H. Gutiérrez, William D. Martin, Chris Bailey-Kellogg, Frances Terry, Leonard Moise, Anne S. De Groot

**Affiliations:** 1 Institute for Immunology and Informatics, CMB/CELS, University of Rhode Island, Providence, RI 02903 USA; 2 EpiVax, Inc., Providence, RI 02860 USA; 3 Department of Computer Science, Dartmouth College, Hanover, NH 03755 USA

**Keywords:** PigMatrix, EpiMatrix, Computational vaccinology, Epitope prediction, HLA, SLA, MHC, Class I, Class II, Porcine, PRRSV, Influenza, Genome-derived vaccine, T cell epitope

## Abstract

**Background:**

T cell epitope prediction tools and associated vaccine design algorithms have accelerated the development of vaccines for humans. Predictive tools for swine and other food animals are not as well developed, primarily because the data required to develop the tools are lacking. Here, we overcome a lack of T cell epitope data to construct swine epitope predictors by systematically leveraging available human information. Applying the “pocket profile method”, we use sequence and structural similarities in the binding pockets of human and swine major histocompatibility complex proteins to infer Swine Leukocyte Antigen (SLA) peptide binding preferences.

We developed epitope-prediction matrices (PigMatrices), for three SLA class I alleles (SLA-1*0401, 2*0401 and 3*0401) and one class II allele (SLA-DRB1*0201), based on the binding preferences of the best-matched Human Leukocyte Antigen (HLA) pocket for each SLA pocket. The contact residues involved in the binding pockets were defined for class I based on crystal structures of either SLA (SLA-specific contacts, Ssc) or HLA supertype alleles (HLA contacts, Hc); for class II, only Hc was possible. Different substitution matrices were evaluated (PAM and BLOSUM) for scoring pocket similarity and identifying the best human match. The accuracy of the PigMatrices was compared to available online swine epitope prediction tools such as PickPocket and NetMHCpan.

**Results:**

PigMatrices that used Ssc to define the pocket sequences and PAM30 to score pocket similarity demonstrated the best predictive performance and were able to accurately separate binders from random peptides. For SLA-1*0401 and 2*0401, PigMatrix achieved area under the receiver operating characteristic curves (AUC) of 0.78 and 0.73, respectively, which were equivalent or better than PickPocket (0.76 and 0.54) and NetMHCpan version 2.4 (0.41 and 0.51) and version 2.8 (0.72 and 0.71). In addition, we developed the first predictive SLA class II matrix, obtaining an AUC of 0.73 for existing SLA-DRB1*0201 epitopes. Notably, PigMatrix achieved this level of predictive power without training on SLA binding data.

**Conclusion:**

Overall, the pocket profile method combined with binding preferences from HLA binding data shows significant promise for developing T cell epitope prediction tools for pigs. When combined with existing vaccine design algorithms, PigMatrix will be useful for developing genome-derived vaccines for a range of pig pathogens for which no effective vaccines currently exist (e.g. porcine reproductive and respiratory syndrome, influenza and porcine epidemic diarrhea).

**Electronic supplementary material:**

The online version of this article (doi:10.1186/s12859-015-0724-8) contains supplementary material, which is available to authorized users.

## Background

The interaction of Major Histocompatibility Complex (MHC) proteins with peptides derived from protein antigens plays a key role in the adaptive immune response mediated by T cells. The MHC:peptide complex presented on the surface of a cell is recognized by the T cell receptor (TCR), which activates the T cell and drives the immune response. There are two classes of MHC molecules: MHC class I presents peptides of intracellular origin to CD8^+^ T cells (cytotoxic T cells, or CTL) and MHC class II presents peptides of extracellular origin to CD4^+^ T cells (T-helper cells, or Th). Both classes of molecules have similar tertiary structure. Class I molecules have a transmembrane (α) chain noncovalently associated with β_2_-microglobulin where the α_1_ and α_2_ domains form the peptide-binding groove; class II molecules have two transmembrane chains (α and β) where the α_1_ and β_1_ domains form the peptide-binding groove. The MHC class I binding groove is closed, which restricts the length of bound peptides to 8–10 residues; the MHC class II binding groove on the other hand, is open, and peptides can extend beyond the ends of the groove, allowing binding of longer and more flexible peptides of variable lengths (typically 13–25 amino acids) [1].

The tertiary structure of MHC molecules is relatively conserved, even across species. For example, crystallographic studies have shown similarity between Human Leukocyte Antigen (HLA; human MHC) and Swine Leukocyte Antigen (SLA; swine MHC) molecules [2]. The SLA-1*0401 class I allele has been crystallized in complex with peptides derived from 2009-pandemic H1N1 (pH1N1) swine-origin influenza A virus (S-OIV) and Ebola virus. A structural comparison revealed that the SLA class I molecule, SLA-1*0401 contains six pockets in its binding groove, similar to HLA class I molecules. The root-mean-squared deviation (RMSD) for all of the Cα atoms in SLA-1*0401 and HLA-A*1101, which has the highest identity with SLA-1*0401 (78 %), was <0.7 Å indicating a similar arrangement of their backbones. Furthermore, three out of 23 influenza SLA-1*0401 binders were identical to previously defined peptides presented by HLA-A*0101 [2]. For SLA class II, no crystal structures are available, but amino acid SLA-DR sequences are highly similar to their human counterparts. For example, the amino acid sequences of SLA-DRB1*0201 and HLA-DRB1*0101 are 79 % identical.

Due to the importance of peptide binding to MHC molecules in the immune response, human T cell epitope prediction tools have been developed based on a range of approaches and are widely used in vaccine development and experimental immunology [3]. The availability of a large and expanding database of validated MHC ligands has contributed to the development of more accurate algorithms. Epitope predictions using these tools reduce the time and effort required to identify T cell epitopes [4]. The number of epitope prediction tools is more limited for pigs due to the paucity of experimental data available. To overcome the lack of quantitative measurements of MHC interaction for a large number of HLA alleles, ‘pan-specific’ methods have been implemented for prediction of T cell epitopes that bind to MHC for which experimental data are limited or not available. Pan-specific methods use experimental binding data and amino acid sequences of multiple MHC alleles to infer binding preferences to uncharacterized MHC molecules. These methods have been used for development of prediction tools for MHC class I [5–7] and II alleles [8–11], but only NetMHCpan has been used for prediction of SLA class I-restricted peptides [12–16]. This method is based on artificial neural networks (ANN) trained using as input a pseudo-sequence composed of the polymorphic residues in the binding groove of a given MHC, a peptide sequence and the experimental affinity data. To our knowledge, there are no *in silico* tools that are available for SLA class II.

Sturniolo et al. first described a method for using existing data to develop new epitope predictors, the pocket profile method, in 1999 [17]. It has been used to develop pan-specific methods for predicting binding of peptides to HLA class I and II alleles [9, 18]. The approach depends on the identification of certain polymorphic regions within HLA molecules that are known to be the areas of contact between peptides and the binding groove of HLA [19–21]. Contact residues from the HLA molecule that bind the R group (side chain) of a specific amino acid within a linear peptide can be considered to form a pocket for that R group. Thus, each ‘pocket’ can also be described in terms of its amino acid binding preferences (‘pocket profile’). The pocket profiles are nearly independent of the remaining binding groove. So this method assumes that two MHC alleles with identical pocket residues will have the same pocket profile. Therefore, given sufficient information about the contact residues of the set of pockets in the binding groove of an MHC and experimentally determined pocket profiles, it is possible to compose predictive matrices *in silico*. The method was originally applied to develop TEPITOPE, an algorithm for prediction of peptide ligands to 51 HLA class II alleles with known pocket residues [17] and then extended to any HLA-DR molecules with similar pockets (TEPITOPEpan) [9]. A similar method has also been used in the PickPocket algorithm for MHC class I prediction [18]. Whereas TEPITOPEpan uses pocket profiles from TEPITOPE, PickPocket generates binding preferences using position-specific scoring matrices (PSSMs) from binding data directly. Although no publications exist using these algorithms for SLA binding predictions, SLA alleles are available for use in the PickPocket server (www.cbs.dtu.dk/services/PickPocket/).

EpiMatrix is a matrix-based algorithm that uses the pocket profile method to predict potential HLA class I and II T cell epitopes. The first version of this algorithm was developed in 1996, and newer versions have been extensively validated *in vitro* in HLA binding assay and human T cell assays and in animal studies using HLA transgenic mouse models [22–26]. For common class II alleles, EpiMatrix appears to predict more accurately than many available epitope-mapping algorithms [27]. Comparative performance for EpiMatrix class I predictions has not been published; however, the tools have been successfully applied to identify class I-restricted T cell epitopes in human pathogens [28–30]. The pocket profile method was used to develop a matrix for a bovine MHC class I allele [31] and in the early 2000s, this method was also used to derive SLA class II prediction matrices from EpiMatrix, but this work was not published.

**Fig. 1:**
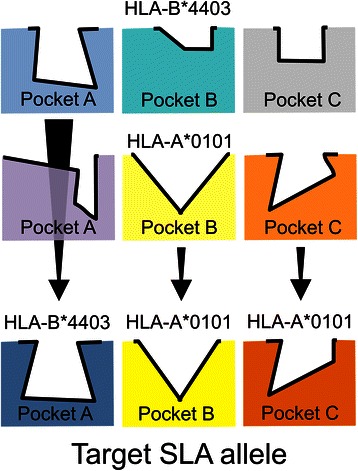
Illustration of PigMatrix development using the pocket profile method. Three pockets (**A, B**, and **C**) from human (HLA) and swine (SLA) MHC molecules are represented as different shapes and colors. The contours of the pockets are shown in bold black lines. HLA pockets from two HLA alleles (HLA-A*0101 and B*4403) are shown in the first two rows. For each pocket in a target SLA, in the third row, we identified the most similar HLA pocket (best human match) and combined their pocket profiles (binding preferences expressed as coefficients) to build composite predictive matrices (PigMatrix)

This paper describes the development and retrospective validation of predictive matrices to map T cell epitopes for SLA class I (SLA-1*0401, 2*0401, 3*0401) alleles and a class II (SLA-DRB1*0201) allele. “PigMatrix” matrices are built based on the pocket profile method using EpiMatrix pocket profiles for HLA epitope prediction. While these alleles represent a small subset of commonly expressed alleles in pigs [32–36], they were selected for their available peptide binding data [13–16, 37–39]. As before, we assumed that predictive matrices developed for HLA alleles should function as reasonable proxies for the prediction of ligands to SLA molecules with similar pocket profiles. Thus, we developed ‘composite matrices’ by selecting the most similar HLA pocket (best human match) for each SLA pocket, and built matrices composed of the corresponding HLA binding preferences (Fig. 1).

Two methods were used to define the pocket contact residues considering different scenarios of availability of SLA crystal structures. In the first scenario, SLA crystal structures were available, so pockets were defined from these structures. In the second, no SLA crystal structures were available; therefore, contact residues were selected based on crystal structures of HLA. We also tested different substitution matrices (PAM and BLOSUM) to score pocket similarity to define the best human match. PigMatrix was benchmarked against existing SLA prediction tools for class I alleles. Benchmarking against other SLA class II predictors was not possible as no other prediction algorithms are available. The results demonstrate the potential of this approach to develop matrices to make accurate predictions for both SLA class I and II alleles for which experimental binding data are limited or even non-existent.

## Methods

### Datasets

Unique 9-mer peptides with reported binding measurement to a specific SLA allele were compiled from the literature into two datasets: one comprising binders and the other, non-binders, for each of three class I (SLA-1*0401, 2*0401, 3*0401) alleles [2, 13–16]. The SLA-1*0401 dataset included 133 binders and 46 non-binders; 2*0401 included 24 binders and 46 non-binders; and 3*0401, 27 binders and 46 non-binders. Twenty-five (14 %) of the SLA-1*0401 peptides were reported by Zhang et al. [2]; the remaining peptides for 1*0401, 2*0401 and 3*0401 were published by Pedersen et al. in different publications [13–16]. For class II, a dataset was created with peptides specific to SLA-DRB1*0201 from the literature [37–39]. This dataset has 33 binders and 171 non-binders. Peptides with contradictory (both positive and negative) results were discarded (Additional file 1). Additionally, we generated a set of 100,000 unique 9-mer peptides from random sequence proteins with the average amino acid frequencies of the proteins in the Swiss-Prot database for use as a control data set, as previously described [31]. The random proteins were generated using the RandSeq tool from ExPASy [40].

### MHC sequences

Complete amino acid sequences from SLA protein sequences, along with HLA class I (HLA-A*0101, A*0201, A*0301, A*1101, A*2402, A*6801, B*0702, B*0801, B*2705, B*3501, B*4403, B*5101) and class II alleles (HLA-DRB1*0101, 0301, 0401, 0701, 0801, 1101, 1301, 1501), were obtained from the IPD-MHC Database (www.ebi.ac.uk/ipd/). It is important to clarify that the HLA alleles for which binding preferences are available in EpiMatrix are families of alleles that share pocket preferences, rather than individual alleles. The alleles represent 12 class I supertypes [41] and eight class II supertypes [42].

### Binding pocket residues

**Fig. 2:**
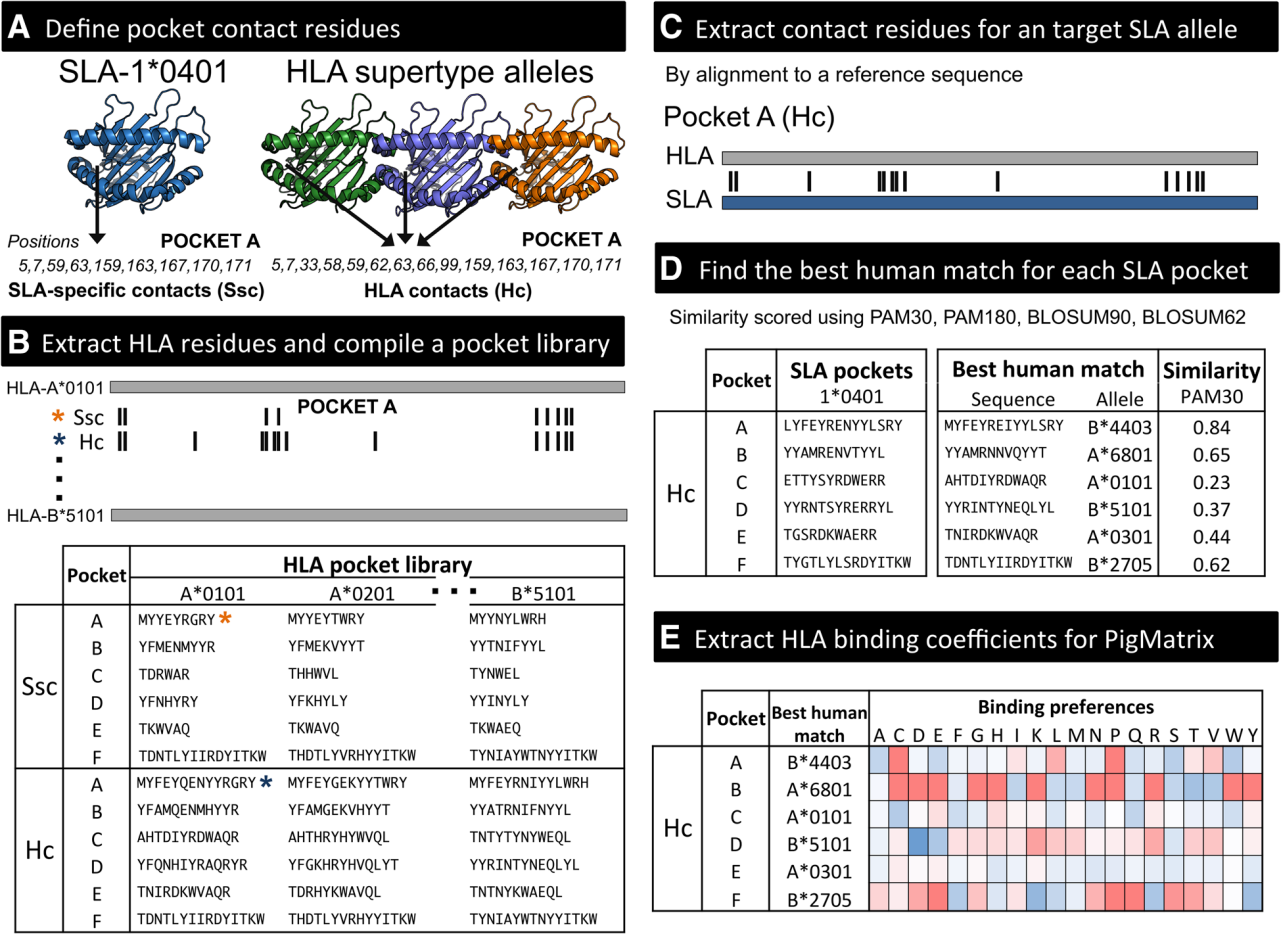
PigMatrix algorithm. **a** Residues in contact with the peptide are determined with respect to a crystal structure of either an SLA allele (Ssc) or an HLA supertype allele (Hc). Class I supertype alleles are represented by three HLA molecules. **b** Contact residues defined by either Ssc or Hc are extracted from HLA sequences and compiled into a library of HLA pockets (HLA pocket library). Pocket A positions and the extracted pocket sequences for Ssc and Hc are marked with *. **c** For a target SLA allele, contact residues (defined by Ssc or Hc) are identified by aligning the SLA sequence to a reference HLA sequence. **d** SLA pocket sequences are compared to those in the HLA pocket library to identify the best human match. **e** Binding coefficients of the best human match for each SLA pocket sequence are compiled to build a PigMatrix. Coefficients are represented in red to blue scale (high to low binding likelihood)

Six pockets (A-F) and five pockets (A-E) were considered for class I and II, respectively. Pockets for peptide positions 4, 5 and 8 for class I and 2, 3, 5, and 8 for class II were not considered due to their minimal effect on binding [17, 19, 43]. For each pocket, contact residues (pocket sequences) were defined as either (1) SLA-specific contacts (Ssc) derived from SLA crystal structures or (2) HLA-based contacts (Hc) derived from HLA crystal structures (Fig. 2a). The Ssc approach was applied only to SLA class I alleles using crystallographic data available for SLA-1*0401 (PDB:3QQ3 and 3QQ4) [2]. For Hc, representative crystal structures from HLA class I and II supertype alleles [41, 42] with bound 9-mer (for class I) or longer peptides (for class II) and the highest resolution were analyzed to define the contact residues (Additional file 2). Four class II supertype alleles (HLA-DRB1*0101, 0301, 0401, and 1501) had crystal structures available.

We considered binding pocket contact residues to be amino acids with atoms within 5.0 Å of those in the bound peptide. Residues were selected using PyMOL (Schrodinger, LLC). Only amino acids with the side chain oriented towards the peptide were included. Thus, each pocket included the union of contact residues in all the MHC crystal structures of a given class. For class II alleles, since the alpha subunit of HLA-DR (HLA-DRA) is practically invariable [44], only residues in the beta subunit were included. The amino acids in the positions defined as contact residues according to Ssc and Hc were extracted from HLA sequences and compiled into a pocket library (Fig. 2b), where each pocket is a non-contiguous sequence of residues ordered by their positions.

### Composite matrix construction

Each SLA protein sequence was aligned to a reference HLA sequence (HLA-A*0101 for class I and HLA-DRB1*0101 for class II) to extract its contact residues (Fig. 2c) based on Ssc and Hc approaches. SLA pockets were compared to the HLA pocket library to identify the best human match for each SLA pocket. SLA-HLA pocket similarity was determined using PAM and BLOSUM substitution matrices for closely (PAM30 and BLOSUM90) and distantly related (PAM120 and BLOSUM62) protein sequences [45, 46]. For a pocket comparison between SLA sequence **x** and corresponding HLA sequence **y**, both of length *N*, the similarity score was calculated as the sum of the similarity scores of each amino acid *i* using a specific substitution matrix *M*. The score was then divided by the similarity score of the SLA pocket compared to itself.

$$ {\mathrm{sim}}_{\mathrm{M}}\left(\mathrm{x},\mathrm{y}\right)=\frac{{\displaystyle {\sum}_{i=1}^N\mathrm{M}\left[{x}_i,{y}_i\right]}}{{\displaystyle {\sum}_{i=1}^N\mathrm{M}\left[{x}_i,{y}_i\right]}} $$

The HLA pocket with the highest similarity score was considered to be the best human match (Fig. 2d). The pocket profiles of the best human matches for each pocket were then combined to form composite matrices (Fig. 2e).

### Matrix validation and performance evaluation

Composite matrices were used to score a set of random 9-mer peptides. The raw binding score bind_raw_(**p**) for each peptide **p** was calculated as the sum, over a set *I* of relevant peptide positions, of the coefficient *K*[*i*, *p*_*i*_] of the amino acid *p*_*i*_ at position *i* in **p**. Positions 1, 2, 3, 6, 7, and 9 were used for class I and 1, 4, 6, 7, and 9 for class II, as those positions most interact with each SLA pocket.

$$ {\mathrm{bind}}_{\mathrm{raw}}\left(\mathrm{p}\right)={\displaystyle {\sum}_{i\in l}K\left[i,{p}_i\right]} $$

The average *μ* and standard deviation *σ* of the scores were used to normalize scores into a Z-score scale (binding likelihood score).

$$ Z=\frac{{\mathrm{bind}}_{\mathrm{raw}}\left(\mathrm{p}\right)-\mu }{\sigma } $$

Next, the ability of the composite matrices to separate binders from non-binders and binders from a set of random peptides was evaluated by comparing the mean of the Z-scores of the datasets [28]. Differences were evaluated for significance by Wilcoxon-Mann–Whitney test. For class II binders longer than 10 amino acids, 9-mers overlapping by eight amino acids were scored because in general, the lengths of MHC binding cores are 9 amino acids [47]. The 9-mer frame with the highest Z-score was selected to be the most likely MHC binder and its score was used for calculation of the mean Z-score of binders and non-binders. In addition, for each allele, the HLA matrix with the lowest overall pocket identity with the SLA was used to score both set of peptides, binders and random peptides, as a negative control matrix.

Peptides in the top 5 % of the normal curve, where the Z-score is greater than or equal to 1.64, were considered to be potential binders. This threshold has been shown to identify peptides that are highly likely to bind HLA molecules [22]. So as to evaluate the predictive performance of the matrices, we calculated the area under the receiver operating characteristic (ROC) curve (AUC) using the sensitivity and 1 - *specificity* values for the same dataset of binders and non-binders.

Finally, PigMatrix SLA class I predictions were compared to those of PickPocket 1.1 and NetMHCpan 2.4 and 2.8. A threshold of 500 nM in binding affinity (or 0.426 prediction score based on 1 − *log*_50*K*_(*affinity*), was set to classify binders and non-binders as previously described [6, 18].

## Results

### Pocket residues

**Fig. 3:**
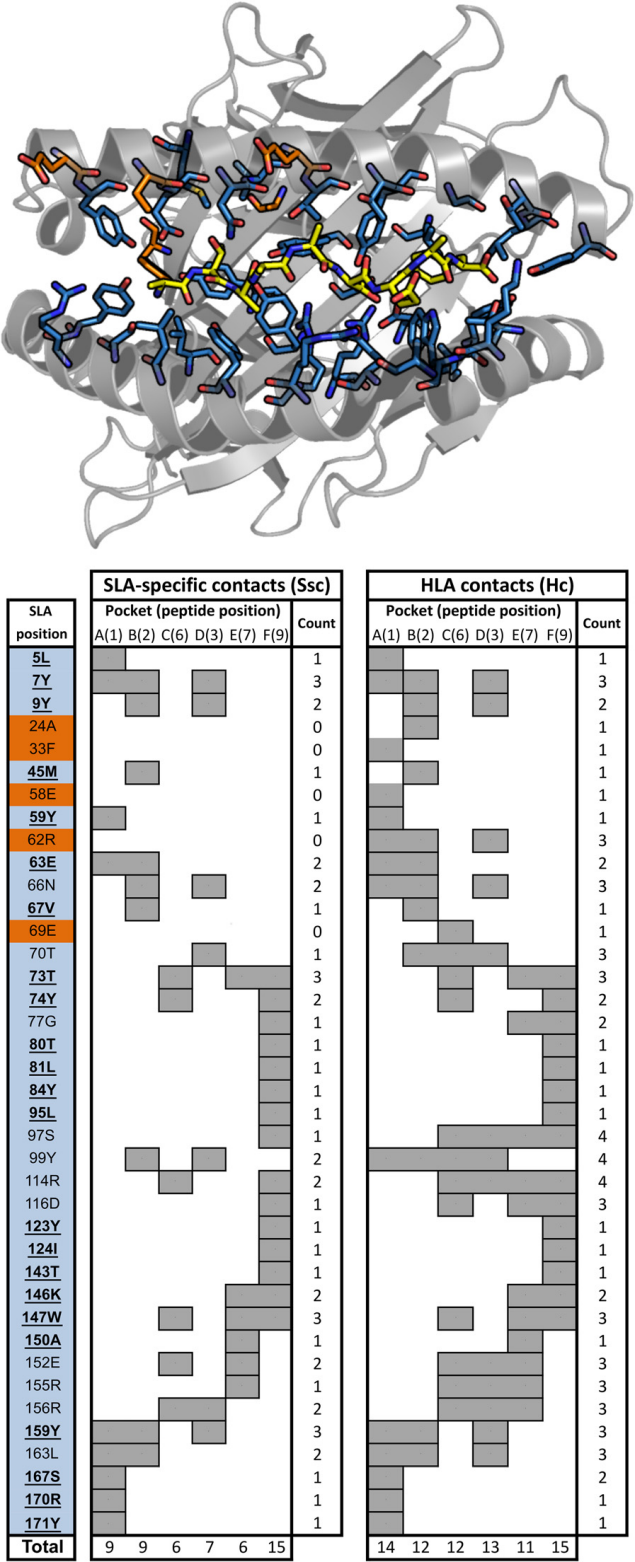
Comparison of contact residues in the binding pockets based on SLA-specific contacts (Ssc) and HLA contacts (Hc).** Top:** Schematic representation of the crystal structure of SLA-1*0401 (PDB:3QQ4; residues 1 to 181 rendered with PyMOL (Schrodinger, LLC)) showing the residues involved in the binding pockets. SLA contact residues and the ligand (ATAAATEAY, yellow) are represented as sticks. Residues common for both Hc and Ssc approaches are show in light blue; residues unique to Ssc in green and unique to Hc in orange. **Bottom:** Positions in the SLA binding pockets are shown. The first column (SLA position) is the residue and position number in the SLA-1*0401 protein sequence (Genbank:2352988). Residue positions shown in bold and underlined are identical (*i.e.* amino acid involved in the same pocket(s)) for both approaches. Positions in light blue are common for both approaches; positions in orange are unique to Hc. The next columns show, shaded in gray, the positions involved in pockets A through F that interact with relative ligand positions (peptide position). The last column (Count) is the number of pockets in which an amino acid participates. The last row (Total) is the total number of residues in each pocket

The contact residues that form the binding pockets in SLA were defined from (1) SLA-1*0401 crystal structures (SLA-specific contacts, Ssc), and (2) HLA class I and II crystal structures (HLA contacts, Hc). Figure 3 shows contact residue similarities and differences for SLA-1*0401 in determinations using Hc and Ssc. Thirty-nine positions were identified with Hc, of which 34 were in common with Ssc (shown in light blue in Fig. 3) and five were unique to Hc (in orange); there were no positions unique to Ssc. Several amino acids were involved in more than one pocket; however, this was more frequent for Hc than Scc due to the nature of the approach; only 23 of the 34 common positions belonged to exactly the same pockets by both definitions (positions shown in bold and underlined in Fig. 3). Hc included for each pocket, the union of amino acids over all the HLA crystal structures analyzed. Based on Hc, positions 97, 99 and 114 were part of four pockets; these residues are located in the central part of the MHC binding groove and depending on the characteristics of their R chain and the bound peptide, they can interact with more than one residue of the ligand. We also observed that the total number of contact residues per pocket was lower in Ssc. The main differences in SLA class I were observed for pockets C and D where SLA structures had fewer contact residues involved in the binding. Pocket F, on the other hand, was identical for both.

For class II, only Hc was applied because no SLA-DR crystal structures were available. Twenty-two positions were considered in contact with ligands; four positions were common to three pockets, four to two pockets and 14 were involved in only one pocket. Five positions were included for pocket A, seven in B, seven in C, eight in D, and seven in F (Additional file 3). In pockets B, C, D, and E, we identified in total 8 allele-specific pocket residues; Y30 in pocket B for HLA-DRB1*1501, D28 and R74 in pocket C for DRB1*0301, Q70 and R74 for DRB1*0301 and R13 for DRB1*1501 in pocket D, and V38 for DRB1*0301 and Y37 for DRB1*0401 in pocket E.

### Matrix construction, validation and evaluation

**Fig. 4:**
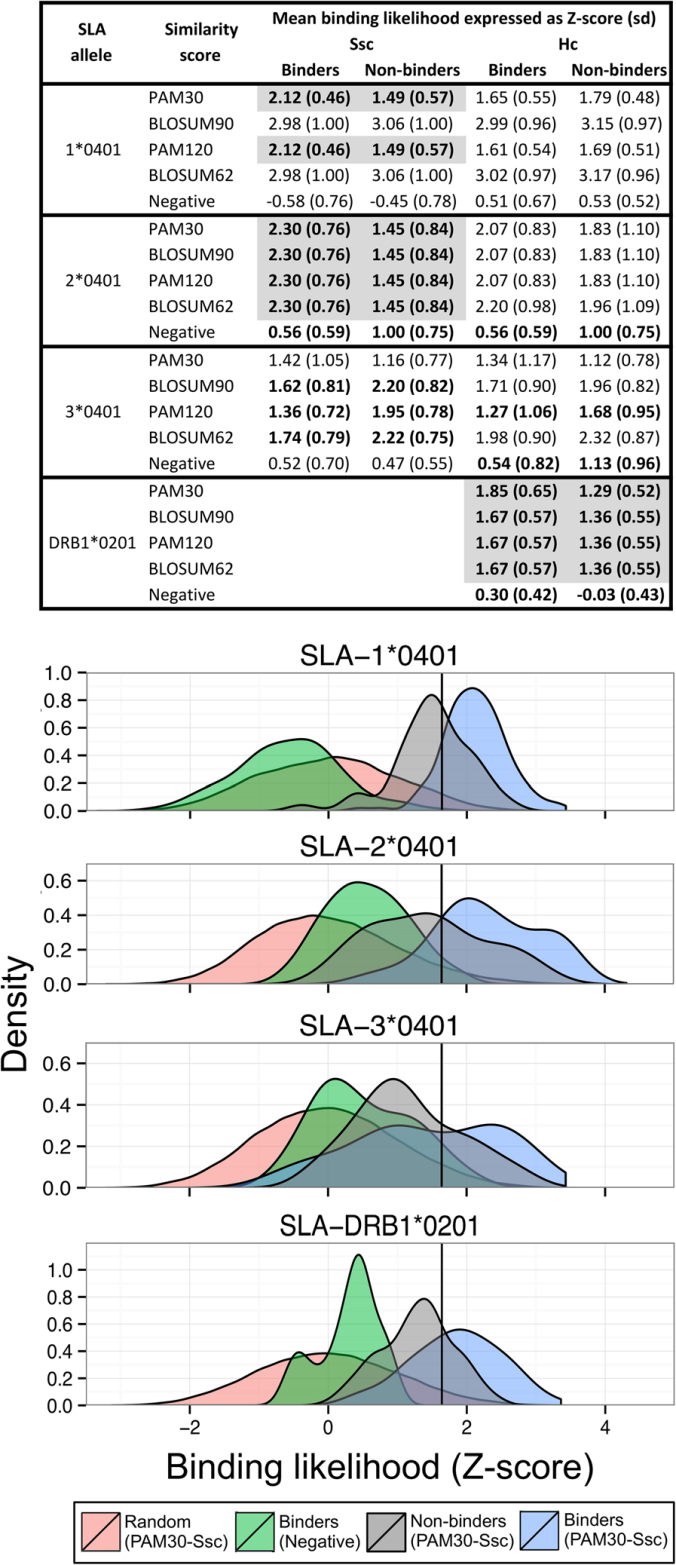
Validation of composite matrices. **Top:** Binding likelihood (Z-score) means and standard deviations (sd) of binders and non-binders calculated using the matrices built based on Ssc and Hc and different scoring methods for pocket selection are shown for SLA class I and II alleles. Z-score means and sd calculated using negative matrices, HLA matrices with the lowest overall pocket identity for each SLA allele, are also shown. Instances where the Z-scores of binders and non-binders were statistically different (*p*-value ≤ 0.05) using a Wilcoxon-Mann–Whitney test, are shown in gray. **Bottom:** Comparison of binding likelihood (expressed as Z-score) between matrices (PAM30-Ssc and Negative) shown as density estimates (smoothed histograms). Note that y-axes are differently scaled. Binders and non-binders were scored with PAM30-Ssc (for class I), PAM30-Hc (for class II) and Negative control matrices. 100,000 natural random 9-mers were scored with either PAM30-Ssc (class I) or PAM30-Hc (class II). The black line indicates the threshold at which a 9-mer is considered a potential binder (Z-score of 1.64). Ssc was not applied to SLA-DRB1*0201 because crystal structures are not available

We built composite matrices for each SLA allele and evaluated whether they were able to distinguish SLA allele-specific binders from non-binders and random peptides (Fig. 4). In some cases, the best human matches were the same regardless of the approach used to define pocket residues and the pocket similarity scoring method applied; therefore, prediction results were identical (e.g. Fig. 4 left, SLA-2*0401). For SLA-1*0401 and 2*0401, two and four scoring methods respectively, generated Ssc matrices capable of separating binders from non-binders (highlighted in gray in Fig. 4 left). For these matrices, mean Z-scores of binders were above the threshold to be considered a potential binder (1.64) and non-binder Z-scores were below. Furthermore, the difference between the sets of peptides was statistically significant (*p* < 0.001) using a Wilcoxon-Mann–Whitney test. None of the class I Hc matrices was able to distinguish with statistical significance binders from non-binders. Likewise, for all SLA-3*0401 matrices, mean Z-scores of the binders were either not above the 1.64 threshold or the non-binders had higher mean Z-scores. For class II allele SLA-DRB1*0201, binders scored using Hc matrices were above the threshold and were statistically distinct from mean Z-scores of the non-binders (p < 0.01). Negative control-matrices for all SLA alleles (using HLA alleles with the lowest overall pocket identity) did not separate binders from random peptides (means range from −0.57 to 1.13), showing that the selection of the best human match based on similarity is critical. In sum, these results show that some composite class I Ssc matrices and class II Hc were able to separate binders from random peptides and non-binders.

**Fig. 5:**
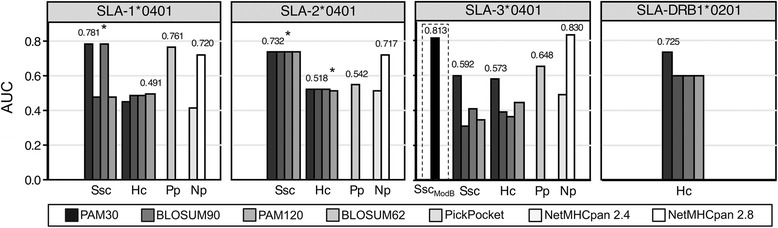
Matrix performance comparison. AUCs of the matrices built for SLA class I and II alleles are shown. The highest AUC for each method is shown above the bars. For SLA-3*0401, AUC of Ssc_ModB_, a PAM30-Ssc matrix with a pocket B profile different than the best human match, is shown in a dashed rectangle to illustrate the impact of pocket B. If one or more matrices for the same approach (Ssc or Hc) have equal AUC, it is indicated with *

To evaluate the predictive performance of the matrices, we built ROC curves and then calculated the AUC. For the AUC, a value of 0.5 corresponds to a random prediction and a value of 1 to a perfect prediction. Figure 5 shows a comparison of the AUCs of Ssc and Hc matrices, PickPocket and NetMHCpan. For class I, matrices based on Ssc had higher AUCs than Hc-based matrices. Class I and II matrices constructed using PAM30 to score pocket similarity had higher AUCs compared to matrices constructed using PAM120, BLOSUM62 and PAM120, with one exception (Hc SLA-1*0401 built using BLOSUM62). Compared to PickPocket and NetMHCpan 2.4 and 2.8, PigMatrix’s AUC was equivalent or better for SLA-1*0401 and 2*0401; however, due to the nature of the tests, we could not assess statistical significance. It is worth noting that, in contrast with NetMHCpan 2.8, SLA peptide data did not contribute to training PigMatrix..

The SLA-3*0401 PAM30-Ssc matrix had the lowest AUC (0.60). This was not unexpected as matrices for this allele were unable to separate binders from non-binders as described above (Fig. 4). For these reasons, we examined the binding preferences of the best human matches for the PAM30-Ssc matrix and compared them to the amino acid frequencies in the sets of binders and non-binders. The most evident differences were observed in pocket B. The best human match for pocket B was HLA-A*0301 (sim_PAM30_ 0.49). Of all binders reported for SLA-3*0401, the most common residue in position two (Pocket B) was arginine, found in 37 % of binders, followed by alanine, found in 19 % of binders. These frequencies were more similar to the binding preferences in HLA-B*2705 (sim_PAM30_ 0.17) pocket profile, in contrast to A*0301, which had negative coefficients for arginine and alanine. Based on this observation, we modified pocket B of PAM30-Ssc matrix from A*0301 to B*2705 (Ssc_ModB_). This matrix had a higher AUC (0.81) than the original matrix (Fig. 5). This result showed that predictions could potentially be improved by selecting best human matches based on similarity in terms of binding preferences if binding information is available. However, it is important to note that this improvement in the AUC was specific to the set of peptides available to date, and further prospective studies are needed to validate the preference of this particular pocket.

Overall, these results demonstrate that matrices built using contact residues from SLA structures and using the PAM30 substitution matrix to identify the best human match for each pocket, had the best predictive power of the approaches that were tested. Although the matrices showed predictive power, the limited number of known binders makes the AUC values less robust. For this reason, an analysis of a larger dataset of SLA-specific binders and non-binder peptides will be required to revalidate the predictive power of the matrices.

## Discussion and conclusions

Immunoinformatics tools have accelerated the identification of epitopes and design of human vaccines. However, comparable tools have not been applied extensively to pigs. During the last two decades, swine T cell epitope discovery has been based on experimental studies of numerous overlapping peptides [37–39, 48–54]. While these studies are essential for validating T cell epitope prediction tools, they can be expensive and time consuming. To reduce experimental effort and expedite the process, algorithms developed for human T cell epitope prediction have also been used to identify porcine epitopes [55–57]. However, the substitution of HLA predictions for SLA predictions may not reflect the fine specificity of SLA binding, which limits the efficacy of this oversimplified approach. To overcome this, we have developed PigMatrix, a simple yet effective method that leverages available data (SLA-binding peptides, SLA structures and HLA binding data) and pocket profiles already constructed for HLA-based epitope prediction in EpiMatrix to predict potential T cell epitopes for SLA class I and II alleles. Using the pocket profile method and the concept that pockets that have similar amino acids will share similar binding preferences, we built and validated matrices that were able to separate SLA-restricted peptides from random peptides and non-binders.

Human pan-specific tools based on the pocket profile method have been described for prediction of class I (PickPocket) and class II T cell epitopes (TEPITOPEpan) [9, 18]. These methods defined the amino acids in the pockets from HLA crystal structures. Similarly, we defined SLA-specific contacts (Ssc) from two crystal structures available for SLA-1*0401. Additionally, we extrapolated the pocket residues from crystal structures of HLA class I and II crystal structures (Hc). Both approaches assume for a given pocket that all contact residues are conserved across all class-specific MHC molecules. However, because there are differences in the pocket residues between MHC alleles and even between the same allele structures depending on the ligand [2], this simplification is a limitation of the peptide:MHC modeling approach. Even so, it is a reasonable approximation when structural information is limited. For class I, differences in the pocket residues using the Hc and the Ssc approaches were noticeable and impacted the subsequent selection of the best human match to build the prediction matrices. Matrices based on pockets defined from SLA structure-specific contacts performed better than HLA-derived pockets. While a definition of the contact residues based on several HLA structures account for the intra- and inter-allelic variability of binding pockets, it also dilutes the importance of key residues in the peptide:MHC interaction. We speculate that more allele-specific pockets could potentially improve the selection of the best human match and therefore the predictions. Selection might be also improved by weighting the similarity score by conservation of key contact residues.

PickPocket and TEPITOPEpan use a method based on BLOSUM62 to calculate a weighted score of specificity to define the most similar HLA-derived pocket. For PigMatrix, in addition to BLOSUM62, we used PAM120, which is considered equivalent to BLOSUM62 for comparison of distantly related proteins [46], to calculate pocket similarity. We also included PAM30 and BLOSUM90, which are both designed to score similarity between closely related protein sequences. The SLA matrices with highest AUC were based on PAM30 using both Hc and Ssc, with only one exception. If we consider the pocket contact residues as short pseudo-sequences, the better performance of PAM30-based matrices might be explained because low-numbered PAM matrices are more efficient for searches involving short sequences. BLOSUM62 on the other hand, performs better to identify distant homologs using longer sequences. BLOSUM90, like PAM30, is used for closely related sequences; however, it is not recommended for short peptides [46] and unsurprisingly did not perform as well as PAM30 in these studies.

Predictive methods for porcine T-cell epitopes are limited, and none existed previously for SLA class II. PigMatrix is the first tool to make binding predictions for an SLA-DR allele. Class II predictions were limited to the Hc method because no SLA-DR molecule has been crystallized. Since SLA-DR-specific binding data are scarce, predictions require further prospective validation. While it is not possible at this time to benchmark the SLA-DR matrix against other predictors, a comparison can be made for the SLA class I matrices developed here. NetMHCpan has been used for SLA binding predictions. PickPocket, which is also based on the pocket profile method, has been described primarily for HLA class I [18], but predictions are also available for SLA alleles. In this study, for an existing set of published peptides, PigMatrix performed equally or better than two versions of NetMHCpan and PickPocket for SLA-1*0401 and 2*0401. While PigMatrix and PickPocket derive SLA binding preferences from HLA binding data, NetMHCpan artificial neural networks are trained using information derived from available binding data as well as peptide sequences and MHC sequence information [6]. It was previously demonstrated that in a scenario where the quantitative binding data were limited for human and non-human MHC alleles, PickPocket performed better than NetMHCpan [18]. This is also evident when NetMHCpan 2.4 results are compared to NetMHCpan 2.8 predictions. NetMHCpan 2.4 was trained with a limited set of SLA binders and its predictions were equivalent to random selection (average AUC 0.47). Version 2.8, on the other hand, was trained with more data and its performance improved for the alleles we evaluated (average AUC 0.76). Conversely, PigMatrix was not trained with SLA-specific binding data and performed similarly or better than NetMHCpan 2.8 predictions for two of three class I alleles we tested. Moreover, because the number of published peptides is limited, we were not able to compile a test dataset of peptides known to be different from the training set used by NetMHCpan 2.8. Hence, it is possible that NetMHCpan 2.8 performance was overestimated.

For SLA-3*0401, PickPocket and NetMHCpan 2.8 outperformed PigMatrix. Upon closer analysis, these results provided an example of how PigMatrix could be improved. We were able to build a better performing model by modifying pocket B in the matrix constructed using PAM30-Ssc. This might be explained by the role of the amino acid in position two of the peptide as a binding anchor and its specific interaction with this pocket. It is also worth noting that the HLA pocket library we used was limited to 12 class I and eight class II supertypes alleles available in EpiMatrix. It is possible that pocket sequences from other HLA alleles and their profiles are more similar to SLA pockets. Therefore, if the number of HLA alleles in the library is increased, we might find better human matches for SLA pockets, which could potentially improve matrix performance.

So as to illustrate the PigMatrix approach, we built initially matrices for only three SLA class I alleles and one class II SLA allele for which quantitative binding data were available. These alleles are commonly expressed in different porcine breeds and cell lines for *in vitro* culture [32–36]. However, like HLA, SLA diversity is considerable. These results demonstrate the potential of the approach to be extended to SLA alleles with limited or nonexistent epitope binding data. Thus, future versions of PigMatrix will include a more comprehensive and representative set of matrices for SLA alleles expressed in outbred porcine populations. Moreover, prospective *in vitro* and *in vivo* evaluation of PigMatrix predictions will help to refine the matrices.

We developed the PigMatrix tool with the intent to integrate it into the iVAX toolkit, which is a comprehensive set of tools for computational vaccine design that includes EpiMatrix, Conservatrix, ClustiMer, EpiAssembler, JanusMatrix, and VaccineCAD [58]. When the PigMatrices are used to substitute for HLA matrices (EpiMatrix) in iVAX, all of the existing suite of iVAX vaccine design tools can be used with the SLA epitope predictions, which makes it possible to envision accelerated development of novel T cell epitope-based vaccines or whole subunit vaccines optimized for epitope content that protect against infectious disease in swine.

## Additional files

Additional file 1:
**Peptide database.** Exp: Experimental results; 0: Non-binders, 1: Binder.(DOCX 156 kb)
Additional file 2:
**HLA crystal structures.**

Additional file 3:
**Contact residues in the SLA class II binding pockets based on HLA contacts (Hc).**
