# Supplementary material for: Development and validation of an epitope prediction tool for swine (PigMatrix) based on the pocket profile method
Source: BMC Bioinformatics. 2015 Sep 15;16:290. doi: 10.1186/s12859-015-0724-8 (PMC4570239; doi:10.1186/s12859-015-0724-8)
Supplement: Additional file 1: — Peptide database. Exp: Experimental results; 0: Non-binders, 1: Binder.(DOCX 156 kb) [file 12859_2015_724_MOESM1_ESM.docx]

**Additional file 1**

**Peptide database**

Exp: Experimental results; 0: Non-binders, 1: Binder

| SLA-1*0401 | | SLA-2*0401 | | SLA-3*0401 | | SLA-DRB1*0201 | |
| --- | --- | --- | --- | --- | --- | --- | --- |
| **Sequence** | **Exp** | **Sequence** | **Exp** | **Sequence** | **Exp** | **Sequence** | **Exp** |
| ALTDLGLIY | 1 | AAKHMSNTY | 0 | AMYDPQTYY | 0 | AAAPSTTTALDTTPN | 0 |
| AMYDPQTYY | 1 | ASYQFQLPY | 1 | ASYAAAAAY | 0 | AAFVTNSTVADELGR | 0 |
| ASAAHLAAY | 1 | ATAAATEAY | 1 | FLYPSWSLY | 0 | ADALAPVVVEGERAT | 0 |
| ASFAAQLFY | 0 | ATAVNQECW | 0 | FQMDYSLEY | 0 | ADPEYFDEPPRPELP | 0 |
| ASYAAAAAY | 1 | DTRAIDQFF | 0 | GMFANRWII | 0 | AEWILKTLVNTEHAY | 0 |
| ASYAGAGAY | 1 | ESLLHQASW | 1 | GMFSWNLAY | 0 | AFLICLIKVLRGQIV | 1 |
| ASYGAGAGY | 1 | ESPSSDEDY | 0 | HMMAVTLFY | 0 | AHGRKRIVCRERLFS | 0 |
| ASYQFQLPY | 1 | FAHDDRYLY | 0 | HQYPANLFY | 0 | AHGVFNPEFGPAALS | 0 |
| ATAAATEAY | 1 | FGMPNPEGY | 0 | ITMVNSLTY | 0 | ALKLMEKREYKFTCQ | 0 |
| ATAWRTGGY | 1 | FQMDYSLEY | 0 | KARARLLSM | 1 | ALLALYAAAIAAAPS | 0 |
| ATDFKFAMY | 1 | FSSQLGLFY | 0 | KMFHGGLRY | 0 | ANHCSDAMNIMFEEV | 0 |
| ATEDPSSGY | 1 | FSVPLDEGF | 0 | KNNFWFWEY | 1 | APASPEAGAVSTPPV | 0 |
| ATIMPHNLY | 0 | FTFWTFANY | 1 | KRIRLKHIF | 1 | APGLPWALQGKRRGA | 0 |
| ATTFARFLY | 1 | FTIRDVLAY | 1 | KRMMMNLNY | 1 | AQMHSNNGPQIGSAV | 0 |
| ATVKGMQSY | 1 | HMMAVTLFY | 0 | KSFFSRLNW | 1 | AQYRNVWDVDYSAFD | 0 |
| ATYQRTRAL | 0 | HQYPANLFY | 0 | KSYEHQTPF | 1 | ARAMLALLALYAAAI | 0 |
| AVDVDDGHF | 1 | HSNLNDATY | 0 | KTLKGGWFF | 1 | ASLAHADALAPVVVE | 0 |
| AVEDFLAFF | 1 | HTAEIQQFF | 0 | LNIMNKLNI | 1 | ATLSKNKNCILCTVC | 1 |
| AVEGGLYPV | 1 | HTSALSLGY | 1 | LNWFEIWIV | 0 | AVHSGPDEYRRLFEP | 0 |
| AVSFRNLAY | 1 | IIYYQLAGY | 1 | LSNFMLWQF | 0 | AVSFGCAVFPRAGET | 0 |
| CSDETTLYY | 1 | ISRQIHWCW | 0 | MARWITWAM | 1 | CAVFPRAGETFEVRF | 0 |
| CTDDNALAY | 1 | ISVQPLWEW | 0 | MMHASTSPF | 0 | CDGLCVPPEARLAWS | 0 |
| CTELKLSDY | 1 | ITLKVFAGY | 1 | MQYLNPPPY | 0 | CRYDKDADINVVTQA | 1 |
| CTLNKSHLY | 0 | KSAAIDGEY | 0 | RARKRGITM | 1 | DAADALAPSLRCEAV | 0 |
| DSDDWLNKY | 0 | KSLDNYQEW | 0 | RAYRNALSM | 1 | DAETEGVYTWRVLSA | 0 |
| DTEDNVPPW | 1 | KSWPAAIDW | 0 | RIRAANLPI | 1 | DAMNIMFEEVFNTDF | 0 |
| EIAQHGAWY | 1 | MANIFRGSY | 0 | RIYSHIAPY | 0 | DGLDAMEPDTAPGLP | 0 |
| EISGSSARY | 1 | MLYPRVWPY | 0 | RLASYGLYY | 0 | DHAADTVYHLGACAE | 0 |
| ESDMEVFDY | 1 | MNYAAAAAY | 1 | RLFFIDWEY | 0 | DKGFVLGHSITDVTF | 0 |
| ESENISEPY | 0 | MTAASYARY | 1 | RLRRRRHPL | 0 | DTTPNGGGGGNSSEG | 0 |
| ESSDDELPY | 0 | MTAHITVPY | 1 | RLYPFGSYY | 0 | DYRYAISSTNEIGLL | 1 |
| ETESVNSNY | 1 | MTRGLLGSY | 1 | RMFKRVFNM | 1 | DYTCRLEGLPSQLPV | 0 |
| EVAGAGSGF | 1 | MTRVTNNVY | 0 | RMFLAMITY | 0 | DYYPRRSVRLRWFAD | 0 |
| EVDQTKIQY | 1 | MVASQLARY | 1 | RNMSRIFPY | 1 | EAGAVSTPPVPPPSV | 0 |
| FLYPSWSLY | 1 | MVFQNYALY | 1 | RNNDPTLPY | 0 | EALKPHFKSLGQTIT | 0 |
| FSIPVTFSY | 0 | MYADDTAGW | 0 | RQHPGLFPF | 0 | EAMTNYAKEGIQFMK | 1 |
| FTAMQALDY | 1 | QQYHRFGLY | 1 | RRARYWLTY | 1 | EGFAVCDGLCVPPEA | 1 |
| FTDNNELEF | 1 | QTWHGDAPY | 0 | RRFFPYYVY | 1 | EHPVDAAFVTNSTVA | 0 |
| FTFWTFANY | 1 | QTYMYTGQY | 0 | RRFKYLLNV | 1 | EIRPMEKVRAGKTRI | 0 |
| FTIRDVLAY | 1 | RSVWIPGRW | 1 | RRFNRTKPM | 0 | EKREYKFTCQTFLKD | 0 |
| FTYAPAGMY | 1 | RVYNNTARY | 0 | RRLHRLLLM | 1 | EKVRAGKTRIVDVLP | 0 |
| GSDGGLDDY | 1 | RVYPNPEVY | 0 | RRMATTFTF | 1 | ELSPSPPPTPAPASP | 0 |
| GSQYVSLAY | 0 | RYQAQQVEW | 0 | RRRQWASCM | 1 | ENALLVALFGYVGYQ | 1 |
| GTDSGFAAY | 1 | SAYYLDIGF | 0 | RRSRRSLTV | 1 | ENKRITVEGGMPSGC | 0 |
| GTDSNGMLW | 1 | SLRPNDIVY | 0 | RRVRRRVLV | 0 | EPFRAVCVVRDYYPR | 0 |
| GTEKLTITY | 1 | SSLPSYAAY | 1 | RSFRIHILF | 0 | EPIQLAYNSYETQVP | 1 |
| GTEYRLTLY | 0 | SSMNSDAAY | 0 | RTFDRFFEE | 0 | ERLFSARVGDAVSFG | 0 |
| GTFDLGGLY | 0 | SSNAKNSEW | 0 | RTLDTLALY | 0 | ERVHVMRKTKLAPTV | 0 |
| GTTEVNGLY | 0 | SSNPVMSRF | 0 | RVFKETLFL | 0 | FDEPPRPELPRERLL | 0 |
| GVEPGHAFY | 1 | SSVGVTHGY | 1 | RVFNNYMPY | 1 | FEDTQRYDASPASVS | 0 |
| HIASMRRNY | 1 | SSVSSFERF | 0 | RVFYFAIFY | 0 | FEVRFYRRGRFRSPD | 0 |
| HMMAVTLFY | 0 | STEPPMLNY | 0 | RVRRLNWAA | 1 | FGTHFAQYRNVWDVD | 0 |
| HSDDALFIY | 1 | STFATVLEY | 0 | RVYPNPEVY | 0 | FNTDFGFHPNAEWIL | 0 |
| HTAAPWGSY | 0 | TVYNGTSKY | 0 | RWFVRNPFF | 0 | FQGLFEIPSYRSLYL | 0 |
| HTSALSLGY | 1 | VSIPVTNTW | 1 | RYFTVAFLF | 0 | FRSPDADPEYFDEPP | 0 |
| IAGFIEGGW | 0 | VSRLEHQMW | 0 | SARRRHLVF | 1 | FSEALRPHVYHPAAV | 0 |
| ISAYTHWYY | 1 | VSYAAAAAY | 1 | SMFDSWGPF | 0 | FSSANASLAHADALA | 0 |
| ITAGYNRYY | 0 | VTEPGTAQY | 0 | SQYHRFPIY | 0 | GACAEHPGLLNVRSA | 0 |
| ITDITKYLY | 1 | YANMWSLMY | 0 | SRWSRKMLM | 0 | GCNPDVDWQRFGTHF | 0 |
| ITDITSPLW | 1 | YAQMWSLMY | 0 | SSMNSFLLY | 0 | GDKATAHGRKRIVCR | 0 |
| ITDYIVGYY | 1 | YAYNSSLLY | 0 | TSFASSWIY | 1 | GERATVANVSGEVSV | 0 |
| ITFQSILGY | 0 | YLSGIAQYY | 1 | TTRHRKPTY | 0 | GEVSVRVAAADAETE | 0 |
| ITTFFTFAY | 0 | YSRMLYIEF | 0 | TVFYNIPPM | 0 | GFHPNAEWILKTLVN | 0 |
| IVDCLTEMY | 1 | YSYIFLSSY | 0 | VMFRNASEY | 0 | GGGGGNSSEGELSPS | 0 |
| IVDINVKDY | 1 | YTASVVAAY | 1 | VSYAAAAAY | 0 | GKTRIVDVLPVEHIL | 0 |
| KIAPLMVAY | 1 | YTGPDHQEW | 0 | VTFWGFWLF | 0 | GLAAADAADALAPSL | 0 |
| KLDAWLLPF | 1 | YTIGIGAFY | 1 | YAYNSSLLY | 0 | GLIVDTRDVEERVHV | 0 |
| KLDPTNTLW | 1 | YTITYHDDW | 0 | YMIGYTAYY | 0 | GLSAPPVLFGEPFRA | 0 |
| KMARLGKGY | 1 | YTNPQFNVY | 0 | YRFRFRSVY | 1 | GLSTAENALLVALFG | 1 |
| KMFHGGLRY | 1 | YTSDYFISY | 1 | YSRPWNWTF | 1 | GQTITPADKSDKGFV | 0 |
| KSDGTGTIY | 1 |  |  | YSYIFLSSY | 0 | GVYTWRVLSANGTEV | 0 |
| KSDLQPPNY | 1 |  |  | YTFFFTQYF | 0 | HFKSLGQTITPADKS | 0 |
| KSNRIPFLY | 1 |  |  | YTYATRGIY | 0 | HMDYGTGFYKPVMAS | 0 |
| KSTDSESDW | 0 |  |  |  |  | HPAAVSVRFVEGFAV | 0 |
| KTAVVVTRY | 1 |  |  |  |  | HPGLLNVRSARPLSD | 0 |
| KTFEWGVFY | 1 |  |  |  |  | HSVLGTANAPLSTYE | 0 |
| KVFFGPIYY | 1 |  |  |  |  | ICAAGSFKVTALNVV | 1 |
| LIDGRTSFY | 1 |  |  |  |  | IFSKHKGNTKMSEED | 0 |
| LLDGLLAWY | 1 |  |  |  |  | IGRFCAQMHSNNGPQ | 0 |
| LSDDAVVCY | 1 |  |  |  |  | IGSAVGCNPDVDWQR | 0 |
| LSTASSWSY | 1 |  |  |  |  | ILAIVLVIMATCVYY | 0 |
| LTAHYCFLY | 0 |  |  |  |  | ILSFARRGTIQEKLI | 0 |
| LTDDMIAAY | 1 |  |  |  |  | INTILNNIYVLYALR | 0 |
| LTDSDSPTY | 1 |  |  |  |  | KAILISCISNKWQFI | 1 |
| LTFLDCLYY | 0 |  |  |  |  | KALFRRCAADYASRL | 0 |
| LTMDREMLY | 0 |  |  |  |  | KFTCQTFLKDEIRPM | 0 |
| MADSFKSDY | 1 |  |  |  |  | KGNTKMSEEDKALFR | 0 |
| MIDSDEWVY | 1 |  |  |  |  | KHKVRNEVMVHWFDD | 1 |
| MIEPRTLQY | 1 |  |  |  |  | KKFFLLSSRVKELII | 1 |
| MIGGIGRFY | 1 |  |  |  |  | KKGKNFSFAGTIIEG | 1 |
| MLASIDLKY | 0 |  |  |  |  | KLASSAFSGLFG | 1 |
| MSAIVSCRY | 1 |  |  |  |  | KTLEAILSFARRGTI | 1 |
| MSNEGSYFF | 0 |  |  |  |  | KTLVNTEHAYENKRI | 0 |
| MSSAAHLLY | 1 |  |  |  |  | LAPSLRCEAVWYRDS | 0 |
| MSWESTAEY | 1 |  |  |  |  | LAPTVAHGVFNPEFG | 0 |
| MTAASYARY | 1 |  |  |  |  | LDGPVDYTCRLEGLP | 0 |
| MTAHITVPY | 1 |  |  |  |  | LEGLPSQLPVFEDTQ | 0 |
| MTRGILGSY | 0 |  |  |  |  | LGHSITDVTFLKRHF | 0 |
| MTRGLLGSY | 0 |  |  |  |  | LIKVLRGQIVQGVIW | 1 |
| MTRVLPFTY | 0 |  |  |  |  | LNEGVVLDEVIFSKH | 0 |
| MTRVTNNVY | 0 |  |  |  |  | LVIMATCVYYRQAGP | 0 |
| MTSGSSSGF | 1 |  |  |  |  | LYALRRHYEGVELDS | 0 |
| NADTLCIGY | 1 |  |  |  |  | MEPDTAPGLPWALQG | 0 |
| NIDNMCHLY | 1 |  |  |  |  | MFEEVFNTDFGFHPN | 0 |
| NSDTVGWSW | 1 |  |  |  |  | MMASLARAMLALLAL | 0 |
| NTDAFSREY | 1 |  |  |  |  | MPSGCSATSIINTIL | 0 |
| QIGNIISIW | 1 |  |  |  |  | MRKTKLAPTVAHGVF | 0 |
| QSAANMYIY | 0 |  |  |  |  | MSEEDKALFRRCAAD | 0 |
| QTDNDIWFW | 1 |  |  |  |  | NDWFSKLASSAF | 1 |
| QTDNQLAVF | 1 |  |  |  |  | NGTEVRSANVSLLLY | 0 |
| QTDPLWQKY | 1 |  |  |  |  | NGTVGPEVEAALKLM | 0 |
| QTEENLLDF | 1 |  |  |  |  | NKDPRLNEGVVLDEV | 0 |
| QTNLYNLLY | 1 |  |  |  |  | NNGPQIGSAVGCNPD | 0 |
| QTWHGDAPY | 1 |  |  |  |  | NNIYVLYALRRHYEG | 0 |
| QVSRPMFLY | 1 |  |  |  |  | NPEFGPAALSNKDPR | 0 |
| RADSMMLGY | 1 |  |  |  |  | NSSEGELSPSPPPTP | 0 |
| RIARFHRPY | 1 |  |  |  |  | NSTVADELGRRTRVS | 0 |
| RLASYGLYY | 1 |  |  |  |  | NVRSARPLSDLDGPV | 0 |
| RMFLAMITY | 0 |  |  |  |  | PAALSNKDPRLNEGV | 0 |
| RSADGSPPY | 1 |  |  |  |  | PADKSDKGFVLGHSI | 0 |
| RTDAWSYPV | 1 |  |  |  |  | PASVSWPVVSSMIVV | 0 |
| RTLASGLIY | 1 |  |  |  |  | PDEYRRLFEPFQGLF | 0 |
| RTWAYHGSY | 1 |  |  |  |  | PEVEAALKLMEKREY | 0 |
| RTWFHGSLY | 1 |  |  |  |  | PPPSVSRRKPPRNNN | 0 |
| RTWHYCGSY | 0 |  |  |  |  | PPPTPAPASPEAGAV | 0 |
| RTWNYHGSY | 1 |  |  |  |  | PRNNNRTRVHGDKAT | 0 |
| RVERIKSEY | 1 |  |  |  |  | PVLFGEPFRAVCVVR | 1 |
| RVFPGDHFY | 1 |  |  |  |  | PVMASKTLEAILSFA | 0 |
| RVSTGLYRY | 1 |  |  |  |  | PVVVEGERATVANVS | 0 |
| SSDDIPPRW | 1 |  |  |  |  | QEKLISVAGLAVHSG | 0 |
| SSDISFIKY | 1 |  |  |  |  | RADVPGLAAADAADA | 0 |
| SSDLRSWTF | 1 |  |  |  |  | RAGETFEVRFYRRGR | 0 |
| SSFERFEIF | 0 |  |  |  |  | RCAADYASRLHSVLG | 0 |
| SSMNSFLLY | 1 |  |  |  |  | RCEAVWYRDSVASQR | 0 |
| SSSFSFGGF | 1 |  |  |  |  | RERLLFSSANASLAH | 0 |
| SSVGVTHGY | 1 |  |  |  |  | RHYEGVELDSYTMIS | 0 |
| STAPTGSWF | 1 |  |  |  |  | RIVCRERLFSARVGD | 0 |
| STFATVLEY | 1 |  |  |  |  | RLAWSDHAADTVYHL | 0 |
| STYQPLPLY | 1 |  |  |  |  | RLFEPFQGLFEIPSY | 0 |
| SVAMCRTPF | 1 |  |  |  |  | RPELPRERLLFSSAN | 0 |
| SVDGFRASY | 1 |  |  |  |  | RPHVYHPAAVSVRFV | 0 |
| SVEMNAPNY | 1 |  |  |  |  | RPLSDLDGPVDYTCR | 0 |
| SVEVKLPDY | 1 |  |  |  |  | RQLSSNYILELLYKF | 1 |
| TIDKSSPLY | 1 |  |  |  |  | RRGTIQEKLISVAGL | 0 |
| TLELRSRYW | 1 |  |  |  |  | RSANVSLLLYSQPEF | 0 |
| TMDVNHPIY | 1 |  |  |  |  | RSVRLRWFADEHPVD | 0 |
| TQDLFLPFY | 1 |  |  |  |  | RTRVHGDKATAHGRK | 0 |
| TSDGFINGW | 1 |  |  |  |  | RTRVSVVNVTRADVP | 0 |
| TSSARSSEW | 0 |  |  |  |  | RVAAADAETEGVYTW | 0 |
| TTSDFFVNY | 0 |  |  |  |  | RVLSANGTEVRSANV | 0 |
| TVYNGTSKY | 1 |  |  |  |  | RWFADEHPVDAAFVT | 0 |
| VAGGTGSVY | 1 |  |  |  |  | RYDASPASVSWPVVS | 0 |
| VLDKWNTNY | 1 |  |  |  |  | SATSIINTILNNIYV | 0 |
| VSALRLFNY | 0 |  |  |  |  | SEGKIRQLSSNYILE | 1 |
| VSDGGPNLY | 1 |  |  |  |  | SLLLYSQPEFGLSAP | 0 |
| VSDGPPTGY | 1 |  |  |  |  | SMIVVIAGIGILAIV | 0 |
| VSFNQNLEY | 0 |  |  |  |  | SQLPVFEDTQRYDAS | 0 |
| VSYAAAAAY | 0 |  |  |  |  | SQPEFGLSAPPVLFG | 0 |
| VTDPGGLYY | 1 |  |  |  |  | SQSPYVVVATNAIES | 1 |
| VTIGNAYIY | 0 |  |  |  |  | SRRKPPRNNNRTRVH | 0 |
| VTRGAVLMY | 0 |  |  |  |  | STPPVPPPSVSRRKP | 0 |
| VVAANRSAF | 1 |  |  |  |  | SVAGLAVHSGPDEYR | 0 |
| VVDALRNIY | 1 |  |  |  |  | SVRFVEGFAVCDGLC | 1 |
| WSQDPTMLY | 0 |  |  |  |  | SYFQQYMLKGEYQYW | 1 |
| WTGMVDGWY | 0 |  |  |  |  | TANAPLSTYEAIKGV | 0 |
| WVAGVQLLY | 0 |  |  |  |  | TASALYLISYYVIPQ | 1 |
| YAQMWTLMY | 1 |  |  |  |  | TDVTFLKRHFHMDYG | 0 |
| YIFFASFYY | 0 |  |  |  |  | TEHAYENKRITVEGG | 0 |
| YLSGIAQYY | 1 |  |  |  |  | TFLKDEIRPMEKVRA | 0 |
| YSAEALLPY | 1 |  |  |  |  | TGFYKPVMASKTLEA | 0 |
| YSYIFLSSY | 0 |  |  |  |  | TLILILPTYELTKLY | 1 |
| YTASVVAAY | 1 |  |  |  |  | TRDVEERVHVMRKTK | 0 |
| YTDKIAMSY | 1 |  |  |  |  | TRVWNSASTTAFLIC | 1 |
| YTFEPHYFY | 0 |  |  |  |  | TTLLNGSAFYLVCPI | 1 |
| YTSDYFISY | 1 |  |  |  |  | TTTALDTTPNGGGGG | 0 |
| YTYPCIPEY | 1 |  |  |  |  | TVEGGMPSGCSATSI | 0 |
| YVFVGSSRY | 0 |  |  |  |  | TVYHLGACAEHPGLL | 0 |
| YVFVGTSRY | 1 |  |  |  |  | VALFGYVGYQALSKR | 1 |
| YVYFYDLSY | 1 |  |  |  |  | VANVSGEVSVRVAAA | 0 |
|  |  |  |  |  |  | VASQRFSEALRPHVY | 0 |
|  |  |  |  |  |  | VCVVRDYYPRRSVRL | 0 |
|  |  |  |  |  |  | VDVLPVEHILYTRMM | 0 |
|  |  |  |  |  |  | VDWQRFGTHFAQYRN | 0 |
|  |  |  |  |  |  | VEHILYTRMMIGRFC | 0 |
|  |  |  |  |  |  | VELDSYTMISYGDDI | 0 |
|  |  |  |  |  |  | VELYYKGTTIKLDFN | 1 |
|  |  |  |  |  |  | VLDEVIFSKHKGNTK | 0 |
|  |  |  |  |  |  | VPPEARLAWSDHAAD | 0 |
|  |  |  |  |  |  | VVASDYDLDFEALKP | 0 |
|  |  |  |  |  |  | VVCQSNNKMTDESEY | 1 |
|  |  |  |  |  |  | VVNVTRADVPGLAAA | 0 |
|  |  |  |  |  |  | VWDVDYSAFDANHCS | 0 |
|  |  |  |  |  |  | WALQGKRRGALIDFE | 0 |
|  |  |  |  |  |  | WPVVSSMIVVIAGIG | 0 |
|  |  |  |  |  |  | WYRDSVASQRFSEAL | 0 |
|  |  |  |  |  |  | YAAAIAAAPSTTTAL | 0 |
|  |  |  |  |  |  | YASRLHSVLGTANAP | 0 |
|  |  |  |  |  |  | YDLDFEALKPHFKSL | 1 |
|  |  |  |  |  |  | YEPRDSYFQQYMLKG | 1 |
|  |  |  |  |  |  | YGDDIVVASDYDLDF | 0 |
|  |  |  |  |  |  | YRRGRFRSPDADPEY | 0 |
|  |  |  |  |  |  | YSAFDANHCSDAMNI | 0 |
|  |  |  |  |  |  | YTMISYGDDIVVASD | 0 |
|  |  |  |  |  |  | YTRMMIGRFCAQMHS | 0 |
