# Supplementary material for: Development and validation of an epitope prediction tool for swine (PigMatrix) based on the pocket profile method
Source: BMC Bioinformatics. 2015 Sep 15;16:290. doi: 10.1186/s12859-015-0724-8 (PMC4570239; doi:10.1186/s12859-015-0724-8)
Supplement: Additional file 2: — HLA crystal structures. [file 12859_2015_724_MOESM2_ESM.docx]

**Additional file 2**

**Crystal structures**

| **Allele** | **PDB** |
| --- | --- |
| HLA-A*0101 | 4NQV |
| HLA-A*0201 | 3MRE |
| HLA-A*0201 | 3MRG |
| HLA-A*0301 | 3RL1 |
| HLA-A*1101 | 1X7Q |
| HLA-A*2402 | 1P7Q |
| HLA-A*6801 | 4HWZ |
| HLA-B*0702 | 4U1H |
| HLA-B*0801 | 4QRS |
| HLA-B*2705 | 2A83 |
| HLA-B*3501 | 2CIK |
| HLA-B*4403 | 1N2R |
| HLA-B*5101 | 1E27 |
| HLA-DRB1*0101 | 1T5W |
| HLA-DRB1*0301 | 1A6A |
| HLA-DRB1*0401 | 1J8H |
| HLA-DRB1*1501 | 1YYM |
