# Supplementary material for: Development and validation of an epitope prediction tool for swine (PigMatrix) based on the pocket profile method
Source: BMC Bioinformatics. 2015 Sep 15;16:290. doi: 10.1186/s12859-015-0724-8 (PMC4570239; doi:10.1186/s12859-015-0724-8)
Supplement: Additional file 3: — Contact residues in the SLA class II binding pockets based on HLA contacts (Hc). [file 12859_2015_724_MOESM3_ESM.docx]

**Additional file 3**

**Contact residues in the SLA class II binding pockets based on HLA contacts (Hc)**

| **SLA** | **Pocket (peptide position)** | | | | |
| --- | --- | --- | --- | --- | --- |
| **position** | A(1) | B(4) | C(6) | D(7) | E(9) |
| 9H |  |  |  |  |  |
| 11L |  |  |  |  |  |
| 13F |  |  |  |  |  |
| 26L |  |  |  |  |  |
| 28E |  |  |  |  |  |
| 30Q |  |  |  |  |  |
| 37F |  |  |  |  |  |
| 38L |  |  |  |  |  |
| 47Y |  |  |  |  |  |
| 57D |  |  |  |  |  |
| 60D |  |  |  |  |  |
| 61W |  |  |  |  |  |
| 67L |  |  |  |  |  |
| 70Q |  |  |  |  |  |
| 71R |  |  |  |  |  |
| 74E |  |  |  |  |  |
| 78Y |  |  |  |  |  |
| 81H |  |  |  |  |  |
| 85I |  |  |  |  |  |
| 86L |  |  |  |  |  |
| 89F |  |  |  |  |  |
| 90L |  |  |  |  |  |

Positions of the residues in the SLA binding pockets are shown. The first column (SLA position) is the residue and position in the SLA-DRB1*0201 protein sequence (Genbank:61652983). The next columns show, shaded in gray, the positions involved in pockets A through E that interact with specific positions of the peptides (peptide position).
